# Regional Myopericarditis Mimicking Inferior Myocardial Infarction Following COVID-19 Vaccination: A Rare Adverse Event

**DOI:** 10.7759/cureus.41168

**Published:** 2023-06-30

**Authors:** Mohammed E Almalki, Fahad A Alshumrani, Hussam A Almalki, Asim A Saati, Saeed E Alzahrani, Saleh M Khouj

**Affiliations:** 1 Department of Medicine, College of Medicine, Umm Al-Qura University, Makkah, SAU; 2 Department of Interventional Cardiology and Structural Heart Disease, King Abdullah Medical City, Makkah, SAU

**Keywords:** covid-19 vaccination complication, covid-19 vaccination, anomalous left main coronary artery, cardiac biomarker elevation, chest pain, pfizer covid-19 vaccine, myopericarditis

## Abstract

We present the case of a 41-year-old man who developed myopericarditis after receiving the Pfizer COVID-19 vaccine. The patient experienced a sudden onset of chest and abdominal pain 16 days after vaccination. Electrocardiogram findings revealed deep T-wave inversion and minimal ST-segment elevation. Further investigation through coronary artery angiography and computed tomography identified an anomalous left main coronary artery. Magnetic resonance imaging confirmed the diagnosis of myopericarditis. This case highlights the importance of considering myopericarditis as a potential cause of chest pain and elevated cardiac biomarkers following COVID-19 vaccination, particularly in young individuals. Clinicians should be aware of this adverse event and include it in the differential diagnosis for patients presenting with similar symptoms after vaccination.

## Introduction

Myocarditis, or inflammation of the heart muscle cells, can result from infectious, viral, bacterial, protozoal, or non-infectious causes such as immune-mediated myocarditis and toxic myocarditis [1,2]. In Europe and North America, viral infections are the most common cause of myocarditis [2]. Clinical presentation depends on the degree of inflammation in the myocardium and can range from mild symptoms such as chest pain and palpitations with some electrocardiographic (ECG) changes to critical cardiogenic shock [3]. The incidence of myocarditis is 6.1 per 100,000 in men and 4.4 per 100,000 in women, and the mortality rate is 0.2 per 100,000 in men and 0.1 per 100,000 in women [4]. The Centers for Disease Control and Prevention (CDC) reported a possible link between the COVID-19 mRNA vaccine and myocarditis [5]. It mainly affects younger males within a few days of receiving the second dose, with an incidence of about 4.8 cases per one million [5]. A study among 2,000,278 individuals who had received the COVID-19 vaccine showed that 20 individuals developed myocarditis related to the vaccine, approximately one per 100,000, and symptoms usually occurred from three to 10.8 days after vaccine administration [6]. According to a retrospective study in the United States, 23 male patients with an average age of 25 years (age range: 20-51 years) who were previously healthy (except for one) presented with acute onset of chest pain and high troponin levels within a few days after receiving the vaccine. Most of them had received the second dose. In addition, most of them showed abnormal findings on ECG and echocardiogram, including ST-segment elevation, T-wave inversion, and reduced left ventricular ejection fraction (40% to 50%) [7].

Coronary artery anomalies are rare and are usually accidentally discovered during coronary angiography and autopsy [8]. A study of 126,595 patients undergoing coronary arteriography showed that 1.3% (1686 patients) had coronary artery anomalies, and 87% (1461 patients) of them had anomalies of origin [9].

The most dangerous anomaly is the left main coronary artery originating from the right sinus of Valsalva, which increases the risk of sudden cardiac death if the artery has a malignant (inter-arterial) course. However, the most frequent anomaly is the left circumflex artery originating from the right sinus of Valsalva [10,11]. One percent of the overall population has coronary artery anomalies [12], and the anomaly can occur alone or be associated with other congenital cardiac diseases [13]. Most patients are asymptomatic, and some cases could lead to potentially fatal conditions like myocardial infarction, arrhythmia, and sudden death [14]. We report for the first time a case of a congenital cardiac anomaly with myocarditis after the COVID-19 vaccination.

## Case presentation

A 41-year-old man presented to the hospital in 2021, 16 days after receiving the second dose of the Pfizer COVID-19 vaccine. He was recently diagnosed with dyslipidemia and was a former smoker. The patient came to the emergency department with a sudden onset of chest and abdominal pain that started one day prior. The pain was progressive, stabbing in nature, radiating to both shoulders and associated with generalised body aches. It was aggravated by exertion and inspiration. The patient reported no recent history of respiratory illness, diabetes mellitus, or hypertension.

Initial assessment upon arrival revealed a blood pressure of 120/75 mmHg, 97% oxygen saturation in room air, a heart rate of 65 beats per minute, a respiratory rate of 12 breaths per minute, a temperature of 35.4°C, and a body mass index of 23.88 kg/m2. Examination revealed equal bilateral air entry. Table 1 lists the significant laboratory results.

**Table 1:** The patient's laboratory results at the initial assessment

Test	Result	Normal range
Creatinine, serum	69.84	65.42-119.34 µmol/L
Blood urea nitrogen (BUN)	4.25	2.86-8.57 mmol/L
Aspartate aminotransferase (AST)	57 (High)	8.0-48.0 U/L
Alanine aminotransferase (ALT)	39	12-78 U/L
Troponin	9.09 (High)	≤0010 µg/L
Creatinine kinase	344	200-395 U/L
Haemoglobin	152	132-166 g/L
Platelets	417	150-450 10^9^/L
White blood cell count	6.8	3.9-11 10^9^/L
Lactate dehydrogenase	496 (High)	105-333 IU/L

**Figure 1:**
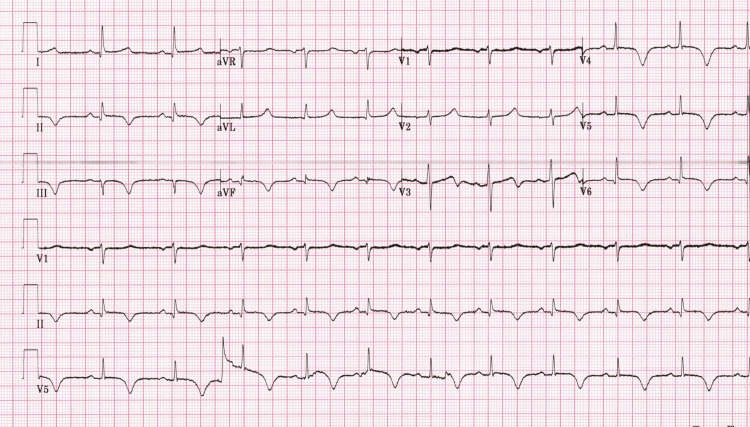
Initial electrocardiogram shows sinus rhythm with deep T-wave inversion in leads III, aVF, and V3–V6, along with minimal ST-segment elevation in the inferolateral leads

An initial ECG showed sinus rhythm and deep T-wave inversion in leads III, augmented Vector Foot (aVF), and V3-V6 with minimal ST-segment elevation in the inferolateral leads (Figure 1).

The patient was referred to a specialised cardiac centre for coronary artery angiography and possible percutaneous coronary intervention. He was admitted to the cardiac care unit as a case of inferior-lateral myocardial infarction for percutaneous coronary intervention. He was vitally stable on admission, and a PCR test for COVID-19 was negative.

**Figure 2:**
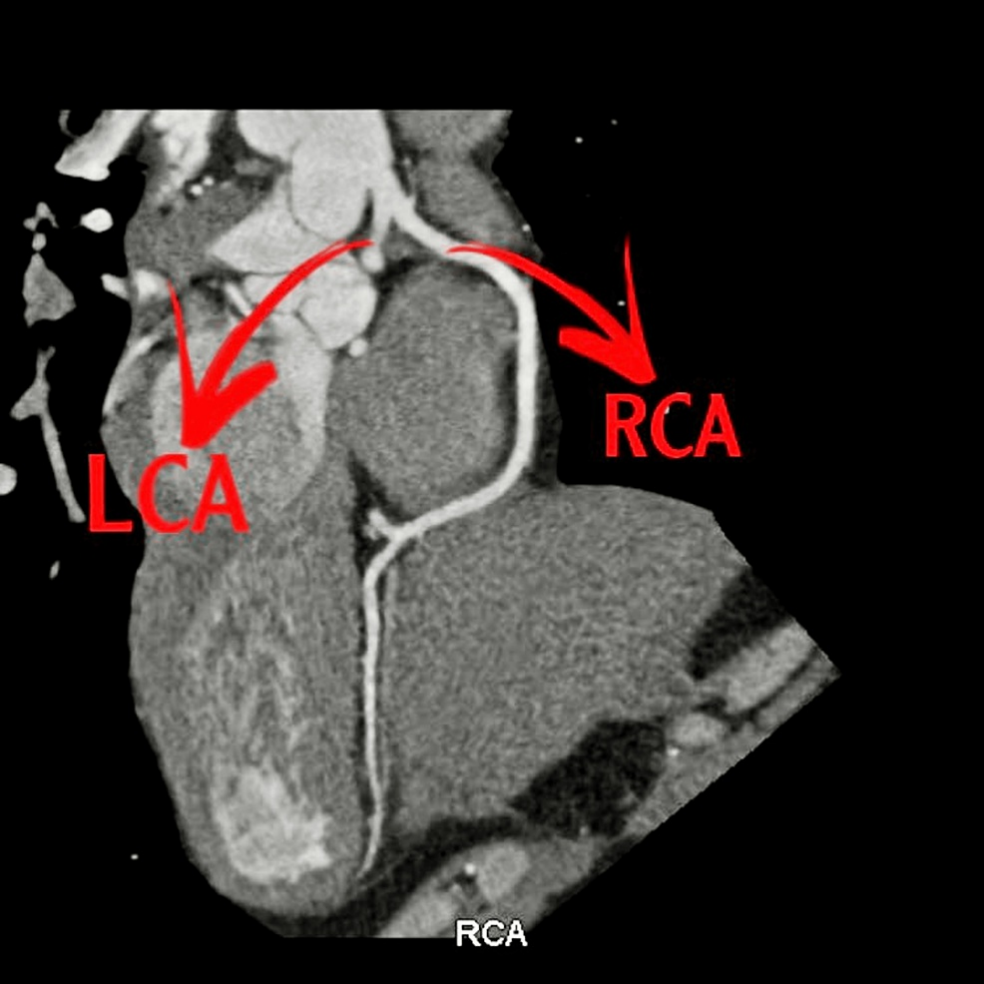
A cardiac computed tomography scan shows bilateral perivascular ground-glass opacities, indicating infection, along with an anomalous left main coronary artery (LCA) originating from the right coronary artery (RCA) with a retro-aortic course

Transthoracic echocardiography showed mild hypokinesia of the inferior wall and normal left ventricular size. Left ventricular systolic function was low-normal. The right ventricle was normal in size and function. The ejection fraction was 50%-55%. There was no intracardiac thrombus, significant valvular lesion, or pericardial effusion. Coronary artery angiography showed an anomalous left main coronary artery arising from the right sinus of Valsalva with no obstructive coronary artery disease. Cardiac computed tomography (CT) showed bilateral perivascular ground-glass opacities highly suggestive of infection, including atypical infection, and an anomalous origin of the left main coronary artery from the right coronary artery with a retro-aortic course (Figure 2).

**Figure 3:**
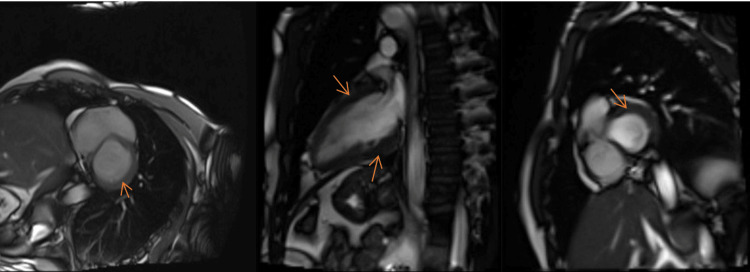
A cardiac MRI demonstrates mildly oedematous mid- to inferior-apical segments with mild hypokinesis and epicardial late gadolinium enhancement, consistent with myopericarditis. Normal left ventricular volume and systolic function are observed, with a left ventricular ejection fraction of 57%.

The patient was moved to the cardiac ward. A cardiac MRI was done and showed mildly oedematous mid- to inferior-apical segments with mild hypokinesis and epicardial late gadolinium enhancement, consistent with myopericarditis, and normal left ventricular volume and systolic function. The left ventricular ejection fraction was 57% (Figure 3).

The final diagnosis was myopericarditis with no obstructive coronary artery disease. The patient was treated with atorvastatin 80 mg once daily, bisoprolol 2.5 mg once daily, and high-dose aspirin, and he improved clinically. He was discharged home in stable condition with close follow-up and an appointment after three months with an echocardiogram to be done before the appointment.

## Discussion

Vaccination is one of the most important types of protection against infection. The COVID-19 vaccine provides effective protection but is not without risk of side effects. Among the potential side effects of COVID-19 vaccines, a documented adverse effect that has been reported in the literature is vaccine-related myocarditis, which may present as chest pain with an incidence of approximately 1.12% [15]. Myopericarditis is an inflammation of the myocardial wall and pericardium caused by a viral infection or autoimmune disease. The patient may present with chest pain, arrhythmia, or heart failure at a late stage. Sudden death may occur in approximately 10% of young adults [16], making early diagnosis crucial. The diagnosis of myopericarditis may be confusing because patients will present with manifestations of acute myocardial infarction. In the case presented, the patient’s ECG showed changes that may be seen in acute myocardial infarction but not in acute myopericarditis, including the presence of pathologic Q waves, a prolonged QT interval, and ST-segment depression in leads facing away from the area of myocardial damage (reciprocal changes). The patient also had an elevated cardiac biomarker; thus, the first impression was to manage it as a case of inferior wall myocardial infarction until cardiac CT and MRI proved otherwise. While there may be some overlap in ECG findings between acute pericarditis and acute myocardial infarction, careful analysis of the ECG can often help distinguish between the two. However, it is important to note that ECG findings alone are insufficient to definitively diagnose either condition; thus, clinical evaluation and additional diagnostic tests may be necessary to confirm.

Myopericarditis can be an adverse effect of vaccines, as a study carried out from 2011 to 2015 of 199 cases of myopericarditis found that 149 of the patients had received the smallpox vaccine [17]. A population study of individuals older than 12 years who can receive the COVID-19 vaccine showed that out of three million vaccination doses administered, 1,626 cases of myocarditis were reported; among them, 1,195 (73%) were over 30 years old. Those younger than 18 years constituted 543 individuals (33%). Symptoms of myocarditis were reported by 82% after the second dose, and 1,334 cases of COVID-19-vaccine-induced myocarditis occurred in males; 291 occurred in females. Consequently, myopericarditis can be triggered by the COVID-19 vaccine. Typical myocarditis can take longer to diagnose than vaccine-induced myocarditis because the symptoms of typical myocarditis may take several weeks to appear after the initial trigger. In contrast, symptoms of vaccine-induced myocarditis may appear within a few days after receiving the vaccine [18]. The clinical course of typical myocarditis can vary widely, with only a small percentage of patients progressing to the point of requiring a heart transplant [19]. This differs from vaccine-induced myocarditis, which has not been associated with documented deaths. Both types, however, show similarities in the predominance of males and in evaluation and treatment [18]. Comparing our case with similar cases reported in the literature, we found consistent clinical features, such as chest pain, and the association between myopericarditis and the COVID-19 vaccines was observed in both our case and others. Notably, the occurrence of myopericarditis in young individuals without known risk factors for coronary artery disease was a shared characteristic (Table 2).

**Table 2:** Comparison between four cases of myopericarditis post the administration of the COVID-19 vaccine ECG: electrocardiography; CT: computed tomography; MRI: magnetic resonance imaging; LVEF: left ventricular ejection fraction; CCU: cardiac care unit; DVT: deep vein thrombosis; sc: subcutaneous; IV: intravenous; ECHO: echocardiography

	Case one (Present case)	Case two [20]	Case three [21]	Case four [22]
Age (in years)	41	24	66	19
Associated vaccine	Pfizer COVID-19 vaccine	Moderna vaccine	Pfizer COVID-19 vaccine	Unspecified mRNA COVID-19 vaccine
First or second dose	Second	Second	Second	First
How long after vaccination did the disease develop?	16 Days	Four days	Three months	14 Days
ECG	Sinus rhythm, T-wave inversion in leads III, AVF, and V3–V6	Sinus rhythm without ischaemic changes	Sinus rhythm with < 1 mm ST elevation in the anterior leads	Normal sinus rhythm with moderate ST depression in V1, V2
Cardiac CT	Anomalous origin of the left main coronary artery. Bilateral perivascular ground-glass opacities were seen.	Small bilateral pleural effusions, without coronary artery stenosis; calcium score of 0		Normal heart size and no pericardial effusion
MRI	LVEF of 57%, mildly oedematous mid- and apical-inferior segments with epicardial gadolinium enhancement	Normal LV size, EF = 58%; mid-myocardial and epicardial delayed gadolinium enhancement, with superimposed oedema	Reduced LVEF (44%) with myocardial and epicardial enhancement along the anterior septum in the mid-ventricular level extending to the base, sparing the subendocardium	
Treatment	The patient was admitted to the CCU and received DVT prophylaxis (enoxaparin 40 mg SC) and IV fluids. The patient was discharged in stable condition with a follow-up appointment after three months and a new ECHO to detect any reduction in LV systolic function.	The patient was discharged from the hospital on beta blockers. He was instructed to avoid strenuous activities for three months as well as non-steroidal anti-inflammatory drugs. A follow-up appointment with cardiology was scheduled.	The patient’s clinical course was significant for the resolution of symptoms within 48 hours of presentation. He was discharged on a beta blocker.	Admitted and given colchicine 0.6 mg twice a day and 81 mg of aspirin. The patient was discharged on day four after clinical improvement.

## Conclusions

We discuss a case of COVID-19 vaccine-induced regional myopericarditis associated with an anomalous left main coronary artery (originating from the right coronary cusp with a retro-aortic course) in a 41-year-old patient complaining of chest pain and body aches after vaccination. Even in light of the small risk of developing a rare case of self-limited myopericarditis, it is recommended to take the COVID-19 vaccine because the benefits outweigh the risks. Future studies should be designed to discover predisposing factors for myopericarditis related to COVID-19 vaccines.
